# Paradoxical Effects of All-*Trans*-Retinoic Acid on Lupus-Like Disease in the MRL/lpr Mouse Model

**DOI:** 10.1371/journal.pone.0118176

**Published:** 2015-03-16

**Authors:** Xiaofeng Liao, Jingjing Ren, Cheng-Hsin Wei, A. Catharine Ross, Thomas E. Cecere, Bernard S. Jortner, S. Ansar Ahmed, Xin M. Luo

**Affiliations:** 1 Department of Biomedical Sciences and Pathobiology, College of Veterinary Medicine, Virginia Tech, Blacksburg, VA, 24061, United States of America; 2 Department of Nutritional Sciences, Pennsylvania State University, University Park, PA, 16802, United States of America; Xavier Bichat Medical School, INSERM-CNRS - Université Paris Diderot, FRANCE

## Abstract

Roles of all-*trans*-retinoic acid (tRA), a metabolite of vitamin A (VA), in both tolerogenic and immunogenic responses are documented. However, how tRA affects the development of systemic autoimmunity is poorly understood. Here we demonstrate that tRA have paradoxical effects on the development of autoimmune lupus in the MRL/lpr mouse model. We administered, orally, tRA or VA mixed with 10% of tRA (referred to as VARA) to female mice starting from 6 weeks of age. At this age, the mice do not exhibit overt clinical signs of lupus. However, the immunogenic environment preceding disease onset has been established as evidenced by an increase of total IgM/IgG in the plasma and expansion of lymphocytes and dendritic cells in secondary lymphoid organs. After 8 weeks of tRA, but not VARA treatment, significantly higher pathological scores in the skin, brain and lung were observed. These were accompanied by a marked increase in B-cell responses that included autoantibody production and enhanced expression of plasma cell-promoting cytokines. Paradoxically, the number of lymphocytes in the mesenteric lymph node decreased with tRA that led to significantly reduced lymphadenopathy. In addition, tRA differentially affected renal pathology, increasing leukocyte infiltration of renal tubulointerstitium while restoring the size of glomeruli in the kidney cortex. In contrast, minimal induction of inflammation with tRA in the absence of an immunogenic environment in the control mice was observed. Altogether, our results suggest that under a predisposed immunogenic environment in autoimmune lupus, tRA may decrease inflammation in some organs while generating more severe disease in others.

## Introduction

Vitamin A plays an important role in the development of a balanced immune system [1, 2]. All-*trans*-retinoic acid (tRA), a predominant vitamin A metabolite, exerts most of the functions attributed to vitamin A [3]. Recent studies of intestinal mucosa have shown that tRA secreted by gut-specific CD103^+^ dendritic cells can modulate the T helper (Th)17-regulatory T cell (Treg) balance [4–6]. tRA has also been shown to induce gut-tropic, IgA-producing B cells [7]. Systemically, tRA is known to regulate Th1-Th2 balance [8, 9] and increase antigen-specific antibody response by promoting the activation and the differentiation of B cells into plasma cells [10–12]. More recently, tRA has been shown to be essential for the differentiation of conventional dendritic cells [13]. These evidences imply that tRA may affect autoimmunity but whether and how tRA, or vitamin A in general, may do so is not clearly understood.

Systemic lupus erythematosus (SLE) is an autoimmune disease with persistent inflammation that damages multiple organs including kidney, skin, lung, heart, joints and brain [14]. A majority of patients are women of childbearing age [14, 15]. SLE is initiated by a breach of immunotolerance to self, which promotes the generation of high affinity autoantibodies primarily against nuclear components and phospholipids [16, 17]. The autoantibodies recognize and bind self antigens, forming immune complexes (IC) that deposit in the peripheral tissues. The complement system is subsequently activated by IC *in situ* and induces inflammation, which amplifies itself by recruiting inflammatory leukocytes [18, 19]. Commonly-used drugs for the treatment of SLE include nonsteroidal anti-inflammatory drugs, antimalarial medicine, glucocorticoids, and immunosuppressive drugs [14]. Recently, several antibody products specifically perturbing autoimmune reactions have been developed to replace the traditional, more toxic chemical agents [14, 20, 21]. However, they may compromise the normal immune response to infections [22, 23]. Therefore, there is a need for new, more natural interventions with minimal side effects.

A beneficial effect of tRA, alone or in combination with low-dose immunosuppressive drugs, on lupus nephritis has been reported in both mouse models and SLE patients [24–28]. However, SLE is a systemic autoimmune disease involving many other organs besides the kidney. Evidence is lacking on how tRA affects other SLE-manifested organs, such as the brain and lung. We herein demonstrate the complex effects of tRA on different peripheral tissues using the classical lupus-prone MRL/lpr mouse model.

## Materials and Methods

### Ethics statement

This study was carried out in strict accordance with the recommendations in the Guide for the Care and Use of Laboratory Animals of the National Institutes of Health. The protocol was approved by the Institutional Animal Care and Use Committee (IACUC) of Virginia Tech College of Veterinary Medicine (Animal Welfare Assurance Number: A3208-01). All animal experiments were conducted under IACUC protocol #12-062. For anesthesia and euthanasia, isoflurane and CO_2_ were used, respectively, according to the IACUC protocol.

### Mice and vitamin A administration

MRL/Mp (MRL) and MRL/Mp-*Fas* ^*lpr*^ (MRL/lpr) mice were purchased from The Jackson Laboratory (Bar Harbor, ME), and bred and maintained in a specific pathogen-free facility following the requirements of Institutional Animal Care and Use Committee (IACUC) at Virginia Polytechnic Institute and State University. All-*trans*-retinoic acid (tRA) and all-*trans*-retinyl palmitate (RP) were purchased from Sigma (St. Louis, MO), and prepared and used in the dark to avoid exposure to light. Both retinoids were dissolved in canola oil (vehicle) and administered orally (directly into the mouth) to female mice from 6 till 14 weeks of age. For tRA treatment, 6 mg tRA/kg body weight (BW) was used per day. This dose was reduced from the reported dose of 10 mg tRA/kg BW [27] that led to skin lesions in MRL/lpr mice in our pilot study, which could affect our analysis of the skin. For daily VARA treatment, 11.2 mg RP/kg BW (equivalent to 6 mg retinol/kg BW) was mixed with 0.6 mg tRA/kg BW (10% of the amount of retinol) before being given to the mice. Mice were weighed twice weekly and the retinoid doses were adjusted accordingly.

### Leukocyte isolation and flow cytometry

For bone marrow cells, bones from both hind limbs of each mouse were cracked gently in a mortar containing phosphate buffered saline (PBS) by using a pestle. Bone marrow was released by gentle stirring after the addition of C10 medium (RPMI 1640, 10% fetal bovine serum, 1 mM sodium pyruvate, 1% 100 MEM non-essential amino acids, 10 mM HEPES, 55 μM 2-mercaptoethanol, 2 mM L-glutamine, 100 U/ml penicillin-streptomycin, all from Life Technologies, Grand Island, NY). The suspension was cleared by passing through a 70-μm sterile cell strainer and carefully layered on the top of Ficoll-Paque Plus (GE Healthcare, Pittsburg, PA). After centrifugation at 1,363×*g* for 30 min at room temperature, mononuclear cells in the buffy coat layer were collected. Spleen and all lymph nodes in the mesenteric region (MLN) were collected and mashed in 70-μm cell strainers with C10. For splenocytes, red blood cells were lysed with RBC lysis buffer (eBioscience, San Diego, CA). For surface marker staining, cells were blocked by anti-mouse CD16/32 (eBioscience), stained with fluorochrome-conjugated antibodies, and analyzed with BD FACSAria II flow cytometer (BD Biosciences, San Jose, CA). For intracellular staining, Foxp3 Fixation/Permeabilization kit (eBioscience) was used. Anti-mouse antibodies used in this study include: B220-FITC, CD3-FITC, I-E/I-A-FITC, CD25-Alexa Fluor 488, CD11c-PE, CD19-PerCP-Cy5.5, CD4-PerCP-Cy5.5, rat IgG2a-APC, CD40-APC, and Foxp3-PE-Cy7 (eBioscience); CD27-PE, Siglec-H-PerCP-Cy5.5, CD138-APC, CD44-APC-Cy7, and CD62L-BV510 (Biolegend, San Diego, CA); mouse IgG2a-PE, I-E^k^-PE, CD11c-PE-Cy7, Ly6C-PE-Cy7, CD11b-APC-Cy7, CD8a-V450, I-A/I-E-V500, and B220-V500 (BD Biosciences); CD3e-APC (Miltenyi Biotec, Auburn, CA). Flow cytometry data were analyzed with FlowJo.

### Enzyme-linked immunosorbent assay (ELISA)

Blood was collected into anticoagulant-coated Capiject tubes (Terumo Medical, Somerset, NJ) and centrifuged at 15,000×*g* for 30 sec. Plasma was collected and stored at -80°C. For detection of anti-double-stranded DNA (dsDNA) IgG, the plate was coated with 0.1 mg/ml of calf DNA (Sigma) in 1 saline-sodium citrate (SSC) buffer at 4°C overnight, followed by washing with PBS containing 0.05% Tween-20 (PBS-T). Wells were then blocked with PBS containing 1% bovine serum albumin (BSA) for 1 h at room temperature and washed. Samples were added and incubated for 1 h at room temperature. After additional washing, HRP-conjugated goat anti-mouse IgG-Fc secondary antibody (Bethyl Laboratories, Montgomery, TX) was added and incubated for 1 h at room temperature, following by more washes with PBS-T. 3,3′,5,5′-Tetramethylbenzidine (TMB) substrate (Biolegend) was then added. After the reaction was stopped, the plate was read at 450 nm with SpectraMax plate reader (Molecular Devices, Sunnyvale, CA). Total IgG and IgM concentrations were determined with mouse IgG and IgM ELISA kits according to the manufacturer’s instructions (Bethyl Laboratories).

### Histology

All fixed tissues were paraffin-embedded, sectioned, and stained for Hematoxylin and Eosin (H&E) or Periodic Acid-Schiff (PAS) at the Histopathology Laboratory at Virginia-Maryland Regional College of Veterinary Medicine. After immersion-fixation in 10% neutral buffered formalin, brains were sectioned in the transverse plane at levels of the following structures: olfactory bulb, head of caudate nucleus, rostral level of hippocampus, caudal level of hippocampus, midlevel of cerebellum with underlying medulla oblongata, caudal level of cerebellum with underlying medulla oblongata. In addition, longitudinal sections of the trigeminal ganglion and adjacent nerve were also obtained. Brain slides were read with Nikon ECLIPSE Ci-L microscope and pictures were taken by using NIS-ElementsD 3.2 64-bit software under 20× objective lense (Nikon Plan 20×/0.40, OFN22 WD1.2) at room temperature. Inflammatory lesions were graded as 0 (no lesions), 1, 2 or 3 (increasing severity of lesions). All brain slides were scored by a board certified veterinary neuropathologist (Jortner) in a blinded fashion. Kidneys were fixed in formalin immediately after isolation, while lung tissues were inflated with formalin through the trachea before submerged in formalin. Lung and kidney slides were read with Olympus BX43 microscope and pictures were taken by using Olympus cellSens software. Lung lesions were scored semiquantitatively (0–4) based on the extent of peribronchial, perivascular, or interstitial lymphocytic infiltration as previously described [29]. Glomerular lesions were graded on a scale of 0–3 for increased cellularity, increased mesangial matrix, necrosis, percentage of sclerotic glomeruli, and presence of crescents [27]. Similarly, tubulointerstitial lesions were graded on a scale of 0–3 for interstitial mononuclear infiltration, tubular damage, interstitial fibrosis, and vasculitis. Slides were scored by a board certified veterinary pathologist (Cecere) in a blinded fashion.

### Immunohistochemistry

Kidneys were embedded in Tissue-Tek O.C.T. Compound (Sakura Finetek, Torrance, CA) and rapidly frozen in a freezing bath of dry ice and 2-methylbutane. Frozen OCT samples were cryosectioned and unstained slides were stored at -80°C. Frozen slides were warmed to room temperature and let dry for 30 min, followed by fixation in -20°C cold acetone at room temperature for 10 min. After washing in PBS, slides were blocked with PBS containing 1% BSA and anti-mouse CD16/32 for 20 min at room temperature. Slides were then incubated with fluorochrome-conjugated antibody mixture for 1 h at room temperature in a dark humid box. Slides were mounted with Prolong Gold containing DAPI (Life Technologies). The following anti-mouse antibodies were used in immunohistochemical analysis: complement C3-PE (Cedarlane, Burlington, NC); IgG-FITC (Sigma); CD11c-PE, CD3e-FITC (eBioscience); and CD138-APC (Biolegend). Slides stained with anti-complement C3 and anti-IgG were read and pictured with EVOS FL microscope (Advanced Microscopy Group, Grand Island, NY) and a 20× objective. Slides stained with antibodies against CD11c, CD3e and CD138 were read and pictured with BX51 upright Olympus microscope (Olympus, Center Valley, PA), a 20× objective and Stereo Investigator software (MBF Bioscience, Williston, VT).

### Reverse transcription-quantitative polymerase chain reaction (RT-qPCR)

Spleen and MLN were homogenized with Bullet Blender homogenizer (Next Advance, Averill Park, NY) and total RNA was extracted with RNeasy Plus Mini Kit (Qiagen, Valencia, CA) according to the manufacturers’ instructions. Genomic DNA was removed by digestion with RNase-free DNase I (Qiagen). Reverse transcription was performed by using iScript cDNA Synthesis Kit (Bio-Rad, Hercules, CA). Quantitative PCR was performed with iTaq Universal SYBR Green Supermix (Bio-Rad) and ABI 7500 Fast Real-Time PCR System (Applied Biosystems, Grand Island, NY). Relative quantities were calculated using L32 as the housekeeping gene. Primer sequences for mouse IL-6, IL-21, IFN, and L32 can be found in S1 Primer Sequences.

### Other measurements

Proteinuria was measured weekly with Chemstrip 2GP (Roche, Indianapolis, IN). A scale of 0–4 was used that corresponded to negative, trace (5–20 mg/dL), 30 mg/dL, 100 mg/dL, and 500 mg/dL total protein, respectively. Dermatitis on the back of the neck and/or face of the mice was observed and recorded in a blinded fashion. Total retinol from liver samples was quantified by Ultra Performance Liquid Chromatography (UPLC) after extraction and saponification. Briefly, portions of each sample (around 0.05 g) were saponified in 5% potassium hydroxide, 1% pyrogallol and 98% ethanol, at 55°C. After extraction into hexanes and phase separation with water, an aliquot of the upper phase lipid extract was mixed with a known amount of internal standard, trimethylmethoxyphenyl-retinol (provided by M. Klaus, Hoffmann-La Roche, Basel, Switzerland). Samples were dried under nitrogen and reconstituted in methanol for UPLC analysis using a C-18 reversed-phase column and mobile phase of 92.5% methanol and 7.5% water at a flow rate of 0.6 ml/min with monitoring at 325 nm. The liver total retinol concentrations were calculated based on areas of the peaks for trimethylmethoxyphenyl-retinol (known amount) and total retinol.

### Statistical analysis

For the comparison of two groups, unpaired student’s *t*-test was used. For the comparison of more than two groups, one-way ANOVA and Tukey’s post-test were used. Results were considered statistically significant when P<0.05. In some experiments, Spearman correlation test and Grubbs’ test for identification of outliers were used. All analyses were performed with Prism software.

## Results

### Identifying an appropriate age of female MRL/lpr mice for intervention

**Fig 1:**
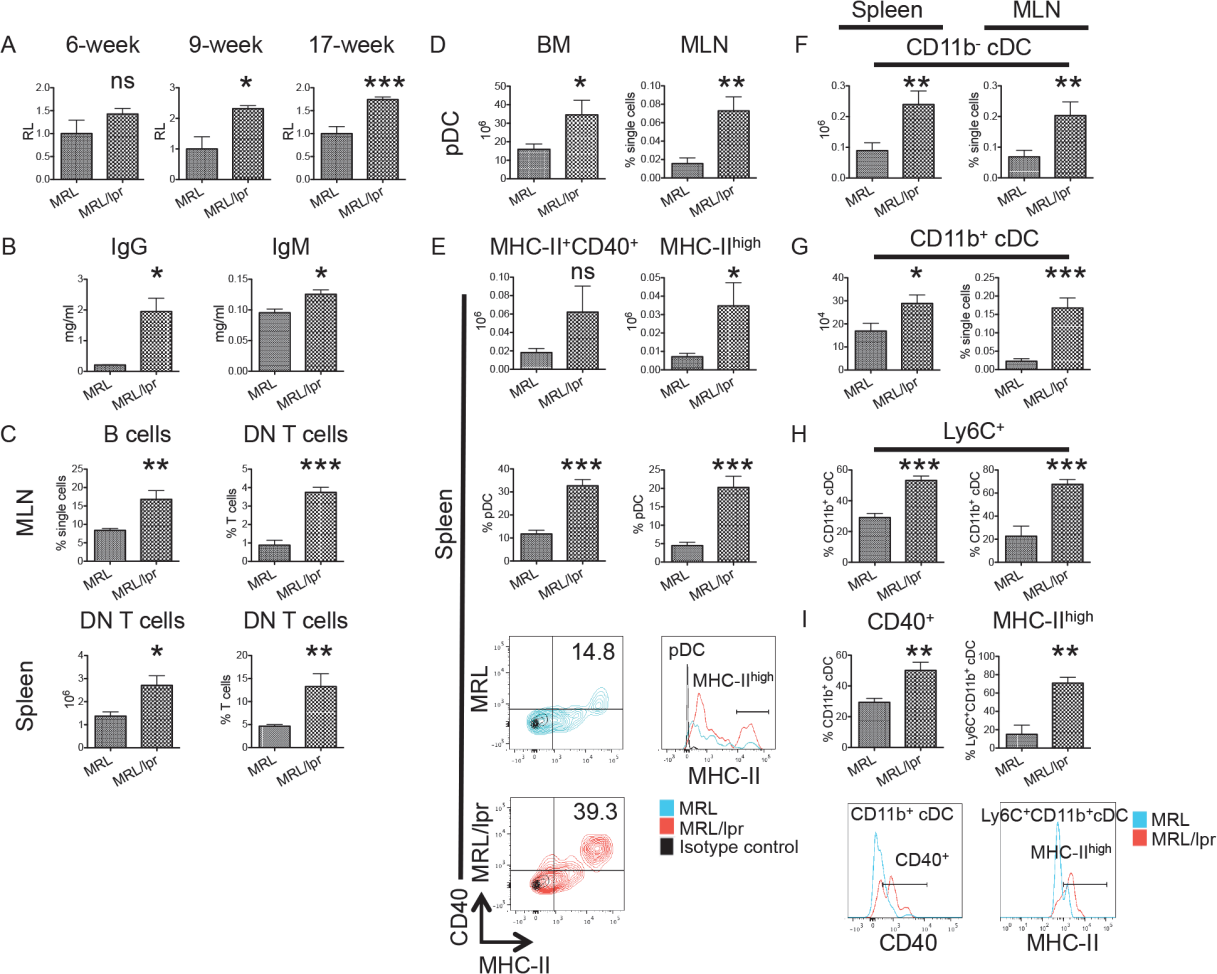
Immunogenic environment in 6-week-old MRL/lpr mice. (A) Anti-dsDNA IgG in the plasma of 6-week-, 9-week- and 17-week-old MRL and MRL/lpr mice as detected by ELISA. Data shown as relative levels (RL) were normalized to the average absorbance value of MRL mice at the same age, which was defined as 1. (B) Total IgG and IgM concentrations in the plasma of 6-week-old mice as detected by ELISA. (C-I) The percentages and absolute numbers of T, B, and dendritic cells in the spleen, mesenteric lymph node (MLN), and bone marrow (BM) of 6-week-old mice as determined by flow cytometry. (C, upper plots) The percentages of B cells (CD19^+^) and CD4^-^CD8^-^ T cells (DN T cells, pre-gated on CD3^+^) in the MLN. (C, lower plots) The absolute cell numbers and percentages of DN T cells in the spleen. (D) pDCs (defined as CD11c^+^CD11b^-^B220^+^Siglec-H^+^) in the BM and MLN. (E) The absolute numbers and percentages of activated pDCs (MHC-II^+^CD40^+^ or MHC-II^high^ pDCs) in the spleen. Representative flow cytometry plots of MRL and MRL/lpr are shown. (F and G) The absolute cell numbers and percentages of CD11b^-^ cDCs (CD11c^+^CD11b^-^B220^-^Siglec-H^-^MHC-II^+^) and CD11b^+^ cDCs (CD11c^+^CD11b^+^B220^-^MHC-II^+^) in the spleen and MLN. (H) Percentages of Ly6C^+^ cells in CD11b^+^ cDCs in the spleen and MLN. (I) The percentage of CD40^+^ cells in CD11b^+^ cDCs in the spleen and the percentage of MHC-II^high^ cells in Ly6C^+^CD11b^+^ cDCs in the MLN. Representative flow cytometry plots of MRL and MRL/lpr are shown. ns: not significant, * P<0.05, ** P<0.01, *** P<0.001, student’s *t*-test. Data are shown as mean + standard error of the mean (SEM), n = 3 mice in each group.

Depending on the immunological state, tRA can promote either immunogenic or tolerogenic immune responses [2]. It is immunosuppressive under steady state [30–32]. However, under an inflammatory environment, evidence has shown that tRA can be immunogenic and deteriorate pre-existing inflammation [4, 33, 34]. Although a beneficial effect of tRA on lupus nephritis has been reported [24–27], whether it would be of benefit or detriment, systemically, to SLE patients with early stages of lupuswhere inflammation has initiated but clinical signs are minimalis unclear. To find an appropriate experimental model to mimic these patients, we first assessed the immunological environment in young, female MRL/lpr mice that were reaching sexual maturity (i.e., around 6 weeks old [35]). We found that, unlike 9- and 17-week-old mice, 6-week-old MRL/lpr mice had a comparable level of anti-dsDNA IgG in the plasma as age-matched MRL controls (Fig. 1A). No kidney pathology was observed, suggesting minimal clinical signs of lupus at this age [27, 36, 37]. However, lymphoproliferation had already initiated as evidenced by higher levels of total IgG and IgM in the plasma of 6-week-old MRL/lpr mice than the controls (Fig. 1B) and accumulations of B cells and double-negative (DN) T in the spleen and MLN of lupus-prone mice (Fig. 1C). These two cell types can contribute to lupus pathogenesis by producing autoantibodies and the proinflammatory cytokine IL-17, respectively [38–42]. Dendritic cells, recently shown to be a strong mediator in lupus development [43, 44], were also investigated. We found that significantly more plasmacytoid dendritic cells (pDCs) were present in the bone marrow and MLN of 6-week-old MRL/lpr mice than age-matched MRL controls (Fig. 1D). In addition, although the number of pDCs in the spleen did not differ, the percentage of splenic pDCs that were MHC-II^+^CD40^+^ or MHC-II^high^ was higher in lupus-prone mice (Fig. 1E), suggesting their activation [45, 46]. Moreover, in both spleen and MLN, accumulation of CD11b^-^ (Fig. 1F) and CD11b^+^ (Fig. 1G) conventional dendritic cells (cDCs) was observed for 6-week-old female MRL/lpr mice. Most accumulated CD11b^+^ cDCs were Ly6C^+^ (Fig. 1H), suggesting that they may have derived from monocytes [47, 48]. Like pDCs, CD11b^+^ cDCs in MRL/lpr mice appear to be activated based on upregulated CD40 and MHC-II expression (Fig. 1I). Taken together, these results suggest that an immunogenic environment has been established in 6-week-old female MRL/lpr mice albeit the lack of overt clinical signs of lupus. Therefore, we decided to investigate the effects of tRA on these mice that mimic patients with early-stage lupus.

### Inflammation of the skin, brain and lung with tRA

To evaluate the effect of tRA on lupus pathogenesis, we treated 6-week-old, female MRL/lpr mice, orally and daily, with vehicle, tRA or VARA till 14 weeks of age. Compared to tRA alone, VARA contained both tRA and retinyl palmitate, the latter being a primary ingredient in vitamin A supplements and thus more clinically relevant than tRA. MRL mice treated with vehicle were used as the control. Retinol analysis showed that vitamin A accumulated in the liver of VARA-treated mice (S1A Fig.). Because tRA is a metabolite of retinol [49–51], tRA-treated mice did not have retinol accumulation in liver. Results of iver function tests (S1B Fig.) and body weight (S1C Fig.) were not different among MRL/lpr groups, suggesting that the administered doses of tRA and VARA were not toxic to the mice.

**Fig 2:**
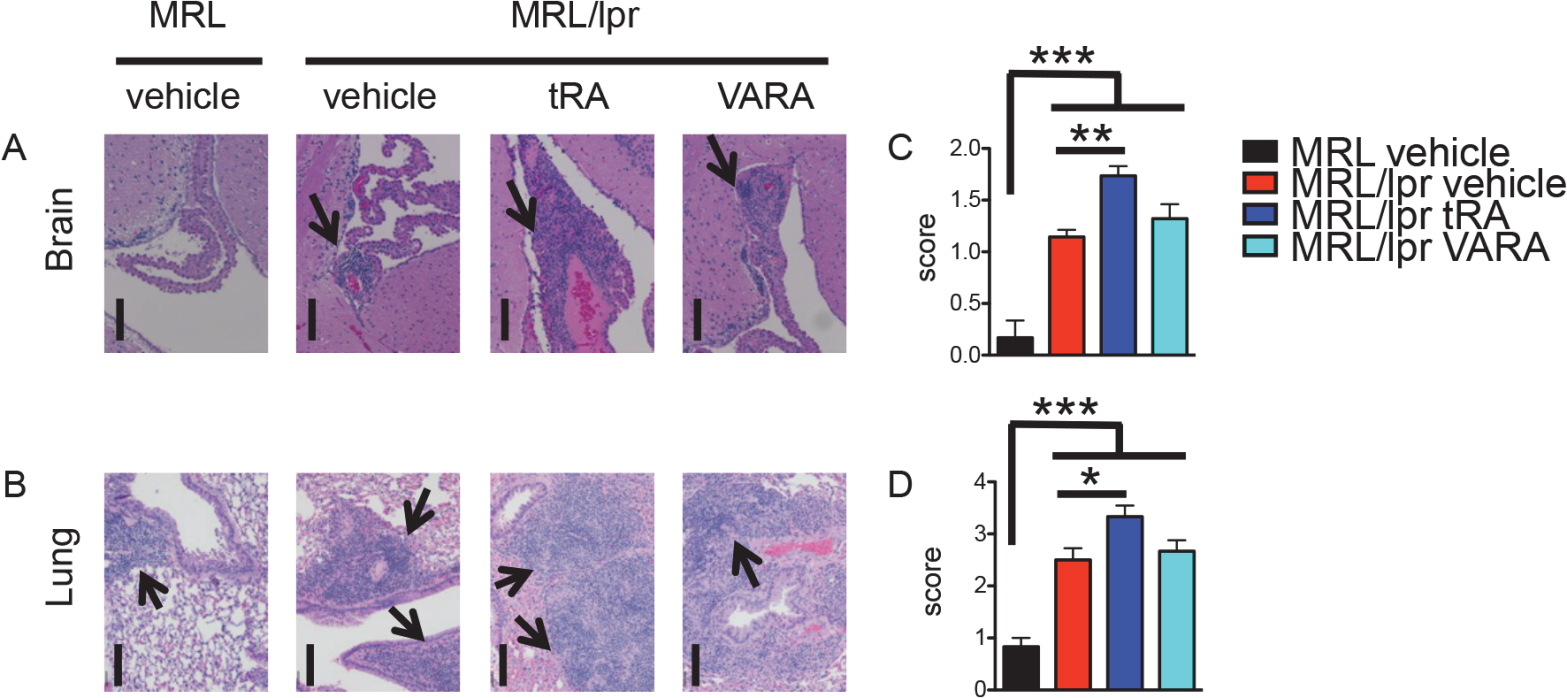
tRA-induced pathology in the brain and lung. Starting from 6 weeks of age, MRL and MRL/lpr mice were given, orally and daily, vehicle (canola oil), tRA (6 mg/kg BW), or VARA (6 mg retinol and 0.6 mg tRA per kg BW) till 14 weeks old when tissues were collected. n = 6 mice in each group. (A) H&E stains of the brain. Representative micrographs are shown. Bar equals 100 m. (B) H&E stains of the lung. Representative micrographs are shown. Bar equals 50 μm. Arrows indicate areas of infiltration. (C-D) Leukocyte infiltration scores of the brain (C) and lung (D) according to H&E stains. * P<0.05, ** P<0.01, *** P<0.001, one-way ANOVA. Data are shown as mean + SEM.

tRA of comparable doses and with similar treatment time frame was previously reported to improve renal pathology in the same mouse model [27]. However, we noted that tRA increased serum concentrations of total antibodies and autoantibodies in the reported study, and wondered if the antibodies would affect the mice at the systemic level, as SLE is a systemic autoimmune disease. Strikingly, we found that tRA worsened lupus-like disease in tissues not investigated in the previous study, including skin, brain and lung. The percentage of mice with dermatitis (S1D Fig.), and the leukocyte infiltration scores of the brain (Fig. 2A and 2C) and lung (Fig. 2B and 2D) increased significantly with tRA treatment compared to MRL/lpr mice treated with vehicle. For the brain, lesions were most profound in the tela choroidea of the 3^rd^ ventricle and adjacent leptomeninges, and in choroid plexus of the 4^th^ ventricle including choroid in the lateral recesses and adjacent leptomeninges. For the lung, although normal peribronchial lymphoid aggregates were present in the MRL control mice, all three MRL/lpr groups exhibited increased severity of peribronchial lymphoid infiltration characterized by mixed small and large lymphocytes and few Mott cells. Perivascular and interstitial infiltrates of lymphoid cells were also observed in the lungs of these mice. In contrast, the effect of VARA on the brain and lung of MRL/lpr mice was comparable to the vehicle control (Fig. 2C and 2D). These results suggest that tRA can increase inflammation in lupus-affected tissues other than the kidney.

### Differential effects of tRA on glomeruli and tubulointerstitium of the kidney

**Fig 3:**
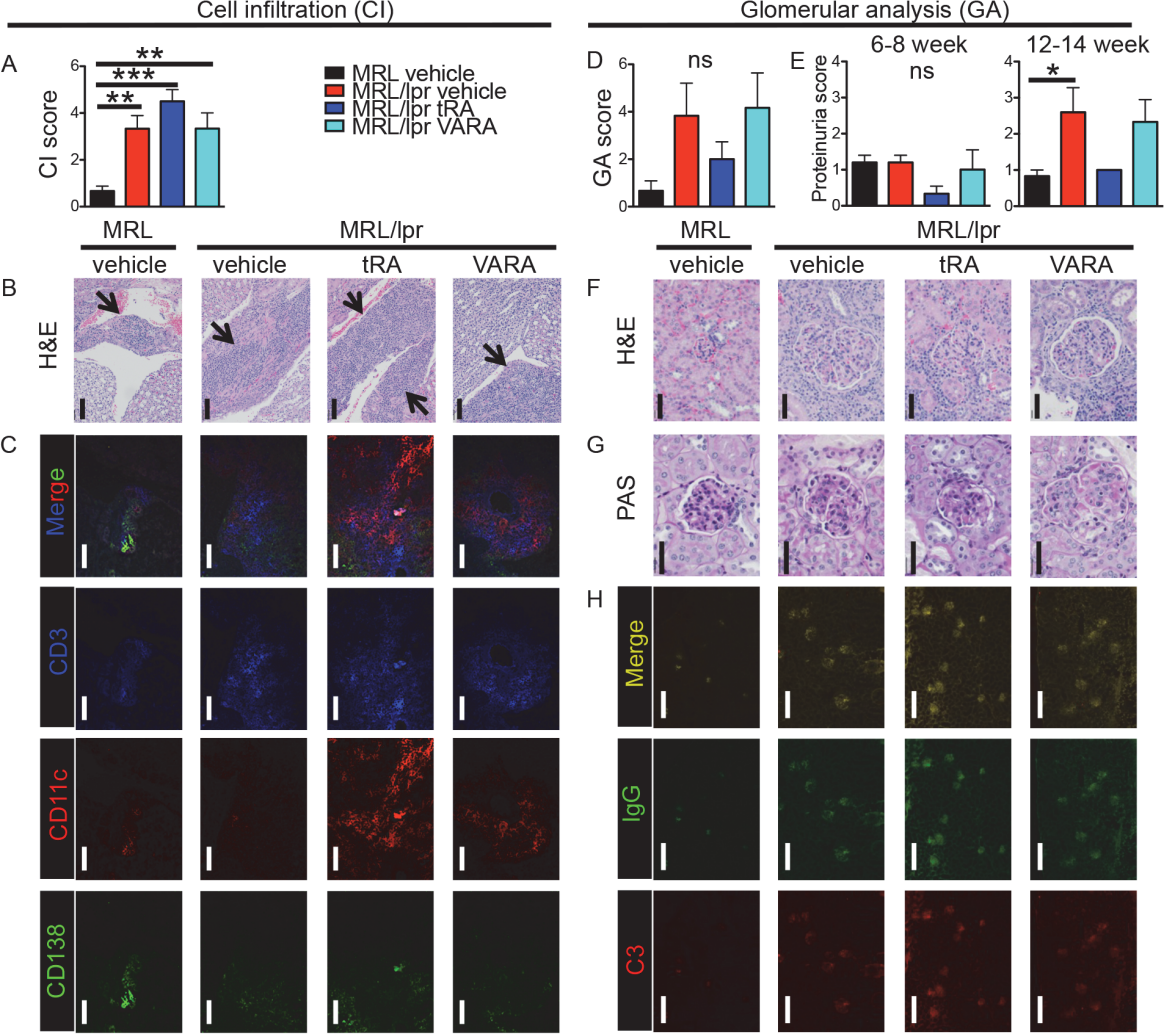
tRA-mediated modulation of kidney pathology. (A-C) Leukocyte infiltration of the tubulointerstitial region. (A) Cell infiltration (CI) scores according to H&E stains. (B) Representative micrographs of H&E stains of the tubulointerstitial region. Bar equals 100 μm. Areas of infiltration are indicated by arrows. (C) Immunohistochemical stains of T cells (CD3-blue), dendritic cells (CD11c-red), and plasma cells (CD138-green). Representative images are shown. Bar equals 100 μm. (D-H) Glomerular analysis (GA). (D) Average GA scores of hypercellularity, mesangial matrix expansion, necrosis, percentage of sclerotic glomeruli, and glomerular crescents. (E) Analysis of proteinuria. The level of total protein in the urine was measured weekly with Chemstrip 2GP. The data of 6- to 8-week-old time points were combined as the early stage, and those of 12- to 14-week-old time points were combined as the late stage. (F) Representative H&E stains showing kidney glomeruli. Bar equals 60 μm. (G) PAS stains showing kidney glomeruli. Bar equals 40 μm. (H) Immunohistochemical stains of IgG (green) and complement C3 (red) deposition in the kidney cortex. Bar equals 200 μm. ns: not significant, * P<0.05, ** P<0.01, *** P<0.001, one-way ANOVA. Data are shown as mean + SEM (n = 6 mice in each group).

Nephritis is one of the most common symptoms in SLE that can cause death [52]. Although the effect of tRA on glomerular pathology had been reported in lupus-prone mice [27], its effect on tubulointerstitium of the kidney was unclear. We found a slight, albeit statistically non-significant, increase of leukocytes infiltrating the tubulointerstitial region of the kidney in tRA-treated MRL/lpr mice compared to lupus-prone mice treated with vehicle (Fig. 3A and 3B). These interstitial lesions contained multiple coalescing perivascular and peritubular infiltrates of mononuclear cells and low numbers of Mott cells. Cellular infiltration was most severe in the medulla but extended into the cortex. In addition, immunohistochemical staining showed the accumulation of a large number of T cells and dendritic cells, and to a lesser extent, of plasma cells, in the interstitium (Fig. 3C). In contrast, although not statistically significant, less severe glomerulonephritis was observed with tRA treatment compared to vehicle-treated MRL/lpr mice (Fig. 3D), which was accompanied by a slight decrease in proteinuria (Fig. 3E). In addition, the size of glomeruli that was enlarged in MRL/lpr mice (as compared to MRL) decreased with tRA treatment (Fig. 3F and 3G), consistent with prior reports [25, 27]. IgG and complement C3 levels in the glomerular region were comparable among the three MRL/lpr mouse groups (Fig. 3H). Taken together, these results suggest that the effect of tRA on the kidney may be region-specific. It increases leukocyte infiltration in the tubulointerstitium while restoring the size of glomeruli in the renal cortex. Treatment with VARA did not affect kidney pathology.

### Reduced lymphadenopathy with tRA

**Fig 4:**
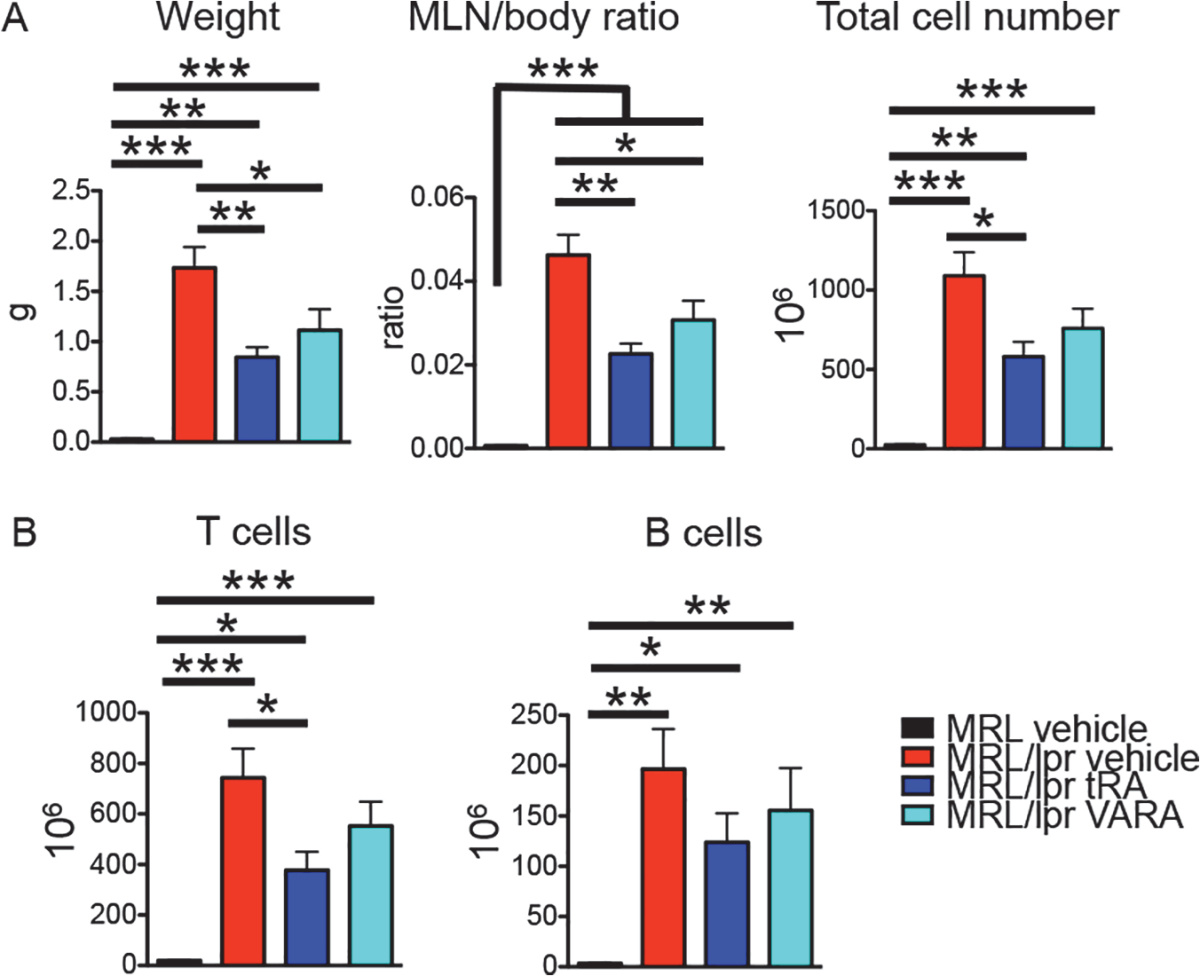
tRA-mediated decrease of lymphocyte accumulation in the MLN. (A) MLN weight, MLN/body weight ratio, and total number of cells in MLN. (B) Absolute numbers of T and B cells in the MLN. * P<0.05, ** P<0.01, *** P<0.001, one-way ANOVA. Data are shown as mean + SEM (n = 6 mice in each group).

**Fig 5:**
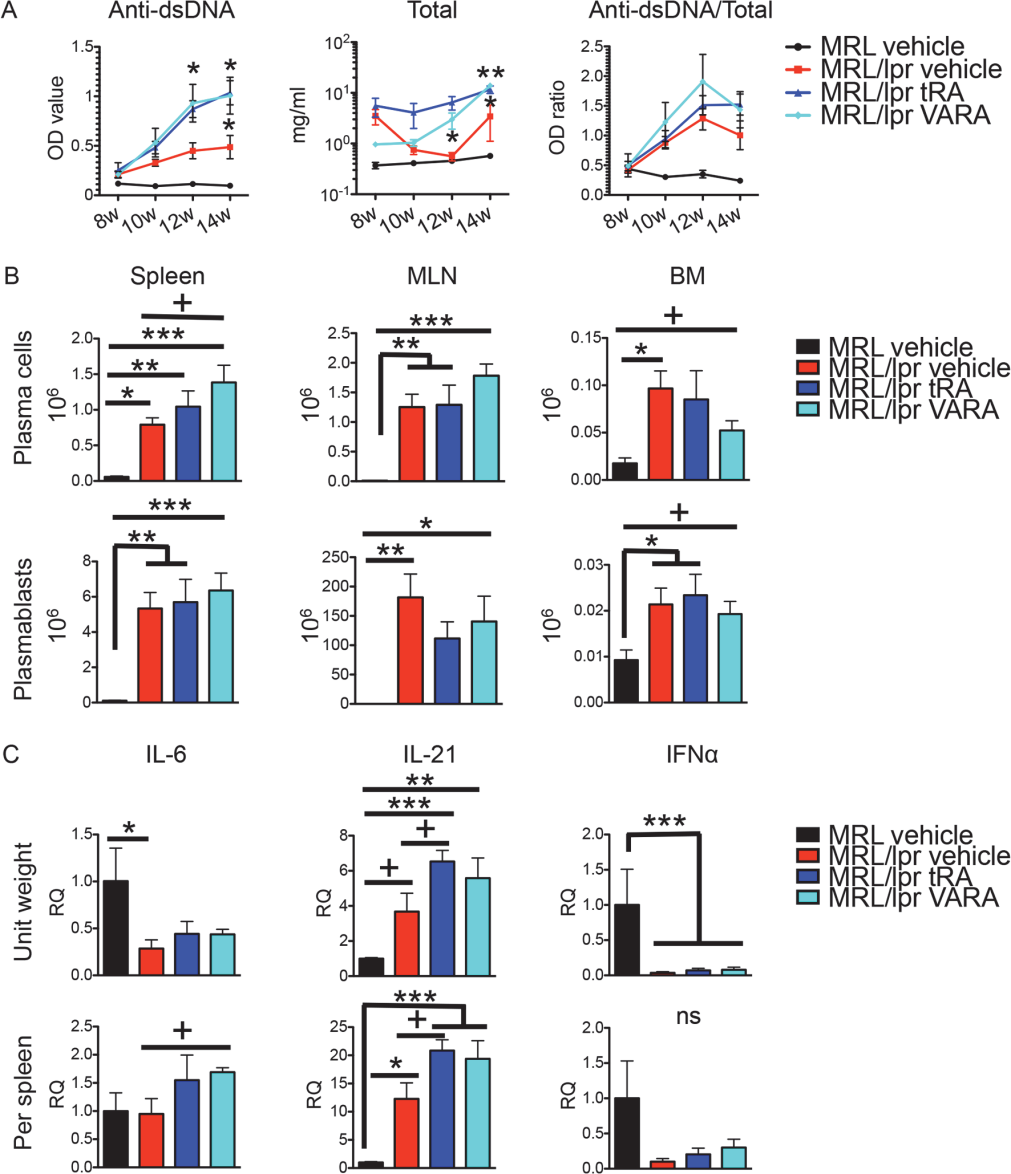
Vitamin A-mediated increase of B-cell responses. (A) Anti-dsDNA IgG, total IgG, and their ratios in the plasma of 8-, 10-, 12- and 14-week-old mice as determined by respective ELISA. One-way ANOVA at each time point was performed but only comparisons between a vitamin A group (either tRA or VARA) and the MRL/lpr vehicle group are labeled in the graphs for simplicity. Although not labeled here, the MRL vehicle group is statistically different from all other groups. Data are shown as mean ± SEM (n = 6). (B) The absolute cell numbers of plasma cells (CD19^-^CD27^-^CD138^+^CD44^+^) and plasmablasts (CD19^+/low^CD27^+/low^CD138^+^CD44^+^) in the spleen, MLN, and BM at 14 weeks old as measured by flow cytometry. (C) mRNA levels of IL-6, IL-21, and IFNα in the spleen at 14 weeks old as determined by RT-qPCR. Relative quantities (RQ) of cytokine mRNA were normalized to that of L32. The average RQ value of MRL vehicle group was defined as 1. ns: not significant, * P<0.05, ** P<0.01, *** P<0.001, one-way ANOVA; +: P<0.05, student’s *t*-test. Except for (A), data are shown as mean + SEM (n = 6 mice in each group).

MRL/lpr mice had significantly larger lymph nodes in the mesenteric region than MRL mice (Fig. 4A and [53]). We found that treatment with tRA significantly decreased the size of MLN in MRL/lpr mice (Fig. 4A), consistent with a prior report [27]. A slight decrease in spleen size was also observed but not statistically significant (S2A Fig.). Closer analysis revealed that the change of MLN size was due to decreased numbers of T cells and B cells, which together represented nearly all mononuclear cells in the MLN (Fig. 4B). VARA exerted similar, but less obvious, effects. Interestingly, the proportions of different T-cell subsets, including CD4^+^, CD8^+^, DN T cells (S2B Fig.) and naïve, central memory, effector memory T cells (S2C Fig.), did not change with tRA or VARA treatment. Because tRA is critical for the generation of gut-tropic Tregs that can suppress intestinal inflammation [31], we examined whether a changed proportion of Tregs in the secondary lymphoid organs accompanied tRA-mediated reduction of lymphadenopathy. It was found that tRA did not change the percentage of Tregs in the spleen or MLN (S2D Fig.). This could be due to the presence of transforming growth factor (TGF) β, a cytokine known to be at an increased level in MRL/lpr mice [54], that was recently shown to suppress tRA-mediated expansion of Tregs from peripheral blood CD4^+^ T cells isolated from SLE patients [55]. In addition, the percentage of activated B cells in the MLN was not affected by tRA, either (see below in Fig. 5B). These results suggest that tRA might have decreased the number of lymphocytes in the MLN without changing their composition or activation. One possible explanation for this phenomenon may be increased trafficking of lymphocytes from MLN to nonlymphoid organs, such as the brain or lung. Dendritic cells, which represented <1% of total cells in the MLN of 14-week-old MRL/lpr mice, were not affected by either tRA or VARA treatment (S2E Fig.).

### Increased circulating autoantibodies with tRA and VARA

We next investigated the humoral immune response that is the cause of type III hypersensitivity in SLE disease [56]. Both anti-dsDNA and total IgG increased significantly in the tRA- and VARA-treated groups compared to the vehicle control in MRL/lpr mice (Fig. 5A). Interestingly, the ratio of anti-dsDNA IgG to total IgG did not change, suggesting that tRA and VARA affected antibody response in general and did not specifically target autoantibodies for expansion. In addition, while neither tRA nor VARA affected the numbers of antibody-secreting B cells in the MLN and bone marrow, the number of plasma cells in the spleen of VARA-treated mice was significantly greater than that of vehicle-treated MRL/lpr mice (Fig. 5B). Moreover, tRA and VARA respectively increase the mRNA levels of IL-21 and IL-6 in the spleen (Fig. 5C). However, another cytokine known to promote plasma cell formation, IFN, was lower in MRL/lpr than MRL mice and did not change with retinoid treatments. This was not entirely surprising because IFNα production had been shown to diminish in old mice with late stage of lupus disease [57]. In the MLN, tRA significantly increased IL-6 and IL-21 mRNA per unit weight but not on the tissue level (S3A Fig.) due to significantly smaller MLN with tRA treatment (Fig. 4A). These results indicate that tRA and VARA increased circulating autoantibodies in MRL/lpr mice, and they might do so through enhancing IL-6/IL-21 production and/or inducing plasma cell differentiation in lymphoid tissues.

**Fig 6:**
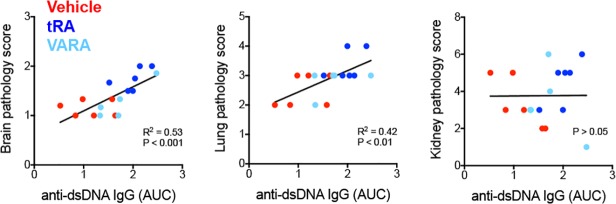
Correlation analysis between blood autoantibody levels and pathological scores. The area under the curve (AUC) was calculated for the level of anti-dsDNA IgG in the circulation and plotted against pathological scores of the brain, lung and kidney. Spearman correlation tests were performed.

To determine whether the increase in circulating autoantibodies correlated with increased pathological scores for the brain, lung and kidney, we performed Spearman correlation analysis (Fig. 6). In the analysis, we excluded one outlier identified by Grubbs’ test (S3B Fig.). It was found that among the 3 organs, the brain pathology score had the strongest correlation with autoantibody accumulation in the blood (R^2^ = 0.53, P < 0.001). The lung had a weaker correlation (R^2^ = 0.42, P < 0.01), whereas no correlation was observed for the kidney. The lack of correlation between kidney pathology and circulating autoantibodies is consistent with recent recognition that pathogenic IgG in the kidney are likely produced *in situ* rather than from the circulation [58–60]. These results and analyses indicate that circulating anti-dsDNA antibodies are detrimental to the brain and lung in lupus-prone mice. It is unclear, however, why VARA increased autoantibody levels in the circulation without affecting brain or lung pathology (Fig. 2). It is possible that the retinol component of the VARA formulation exerted certain protective effects in these organs. This will be investigated in the future.

### Minimal induction of inflammation with tRA in the absence of an immunogenic environment

**Fig 7:**
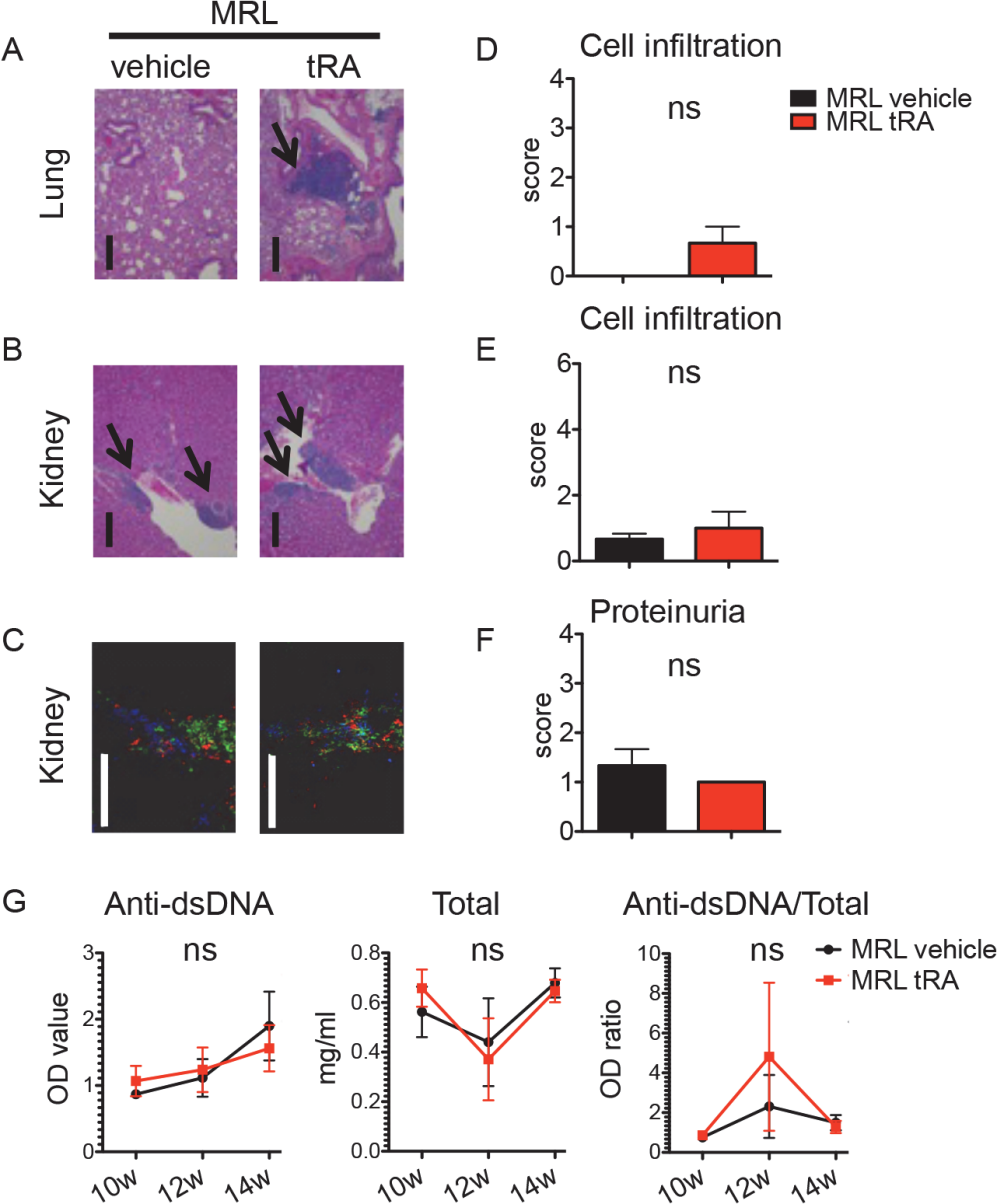
Minimal induction of inflammation with tRA in the absence of an immunogenic environment. MRL mice were given, orally and daily, vehicle (canola oil) or tRA (6 mg/kg BW) from 6 to 14 weeks of age when tissues were collected. n = 3 mice in each group. (A) H&E stains of the lung. Representative micrographs are shown. Bar equals 400 μm. Areas of infiltration are indicated by arrows. (B) Representative micrographs of H&E stains of the kidney. Bar equals 400 m. (C) Immunohistochemical stains of T cells (CD3-green), dendritic cells (CD11c-red), and plasma cells (CD138-blue). Representative images are shown. Bar equals 200 μm. (D-E) Leukocyte infiltration scores of the lung (D) and kidney (E) according to H&E stains. (F) Analysis of proteinuria. The level of total protein in the urine of 14-week-old mice was measured with Chemstrip 2GP. (G) Anti-dsDNA IgG, total IgG, and their ratios in the plasma of 10-, 12- and 14-week-old mice as determined by respective ELISA. ns: not significant, student’s *t*-test. Data are shown as mean + SEM or mean ± SEM.

To evaluate whether the proinflammatory effect of tRA seen in MRL/lpr mice was specific to a predisposed immunogenic environment, we tested the effects of tRA in the absence of such an environment. MRL control mice, which did not display any inflammation at 6 weeks old (Fig. 1), were treated with vehicle or tRA from 6 to 14 weeks of age. Compared to the vehicle group, tRA-treated MRL mice developed dermatitis at the first week of treatment but quickly recovered in the following week. At the end point, there was no dermatitis or leukocyte infiltration in the brain of either vehicle- or tRA-treated mice (data not shown). Minimal amounts of infiltrates were seen in the lung with tRA (Fig. 7A and 7D) and in the kidney for both treatment groups (Fig. 7B and 7E). The cellular composition of renal infiltrates was the same regardless of treatment (Fig. 7C). In addition, the level of proteinuria was not different between the two groups (Fig. 7F). Importantly, while tRA significantly increased the levels of circulating anti-dsDNA and total IgG in MRL/lpr mice (Fig. 5A), it did not do so in MRL mice (Fig. 7G). This was consistent with unchanged or reduced numbers of plasma cells and plasmablasts with tRA in the spleen and MLN (S4A Fig.). Neither did tRA activate T cells in these mice (S4B Fig.). These results suggest that tRA is not a strong inducer of inflammation in the absence of an immunogenic environment.

Taken together, we found in this study that, under a predisposed immunogenic environment in autoimmune lupus, tRA could reduce glomerular injury and lymphadenopathy while generating more severe disease in other lupus-affected organs such as the brain and lung. Administration of this retinoid as a supplement for patients with early-stage lupus should therefore be approached with caution.

## Discussion

A beneficial effect of tRA on lupus nephritis has been reported [24–27]. In lupus-prone MRL/lpr mice, we observed similar decreases in glomerulonephritis (Fig. 3D) and proteinuria (Fig. 3E) with tRA in this study. However, our study extended the investigation to other lupus-affected tissues, as the development of SLE involves multiple organs besides the kidney. Our results showed that although tRA could ameliorate glomerular inflammation, it exerted an opposite effect and worsened inflammation in the skin (S1D Fig.), brain and lung (Fig. 2). In addition, treatment with tRA led to the accumulation of more leukocytes in the renal tubulointerstitium (Fig. 3A). Interestingly, the number of lymphocytes in the MLN decreased with tRA that led to significantly reduced MLN weight (Fig. 4). We hypothesize that this may be due to increased trafficking of lymphocytes from MLN to nonlymphoid organs such as the brain, lung, and tubulointerstitial region of the kidney. Furthermore, we showed that tRA significantly increased the level of circulating autoantibodies (Fig. 5A), which strongly correlated with exacerbated pathology of the brain, and to a lesser extent, that of the lung (Fig. 6). Our results suggest that tRA exerted paradoxical effects on peripheral tissue inflammation in the MRL/lpr mouse model. Table 1 summarizes the changes of all the variables measured in this study.

**Table 1:** Effects of tRA and VARA on MRL/lpr mice.

Variables			Compared to vehicle	
			tRA	VARA
Peripheral tissue pathology	Dermatitis		↑ ^#^	– ^*c*^
	Brain pathology		↑***	–
	Lung pathology		↑*	–
	Kidney pathology	Glomerular score	– (↓)	–
		Leukocyte infiltration (total)	– (↑)	–
		T-cell and DC infiltration	↑ ^#^	–
		Complement/IgG deposition	↑ ^#^	–
		Proteinuria	– (↓)	–
Lymphoid tissues	Spleen	Tissue weight	– (↓)	–
		T cells^*a*^	–	–
		B cells	–	–
		Plasmablasts	–	– (↑)
		Plasma cells	–	↑ *
		Inflammatory cytokines^*b*^	↑ *	↑ *
	MLN	Tissue weight	↓ **	↓ *
		T cells	↓ *	–
		B cells	– (↓)	–
		Plasmablasts	–	–
		Plasma cells	–	–
		Inflammatory cytokines	↑ *	–
Plasma	Anti-dsDNA IgG		↑ **	↑ *
	↑ Total IgG		↑ *	↑ **

^*a*^Changes in the percentage or number of T cells, B cells, plasmablasts, and plasma cells.

^*b*^Transcript levels of IL-21 or IL-6.

^*c*^–: no change

↑: increase

↓: decrease

(↑) and (↓): changes that were not statistically significant

* P<0.05

** P<0.01

*** P<0.001, student’s *t*-test.

^#^ Statistical tests not performed.

tRA is essential for the induction of immune tolerance in steady-state conditions but it can also promote an immunogenic response under inflammatory state [2]. Several lines of evidence in our results support the notion that tRA acts like an adjuvant and facilitates tissue inflammation under an immunogenic environment. First, tRA increased B-cell responses that included more antibody production (Fig. 5A), increased number of plasma cells in the spleen (Fig. 5B), and heightened production of B cell-activating cytokines in MRL/lpr mice (Fig. 5C and S3A Fig.). Second, tRA increased the accumulation of leukocytes in the brain (Fig. 2A), lung (Fig. 2B), and tubulointerstitium region of the kidney in the same lupus-prone mice (Fig. 3A-C), potentially by facilitating leukocyte trafficking into these tissues and promoting local inflammation. Importantly, tRA failed to induce such inflammation in the absence of a predisposed (Fig. 7 and S4 Fig.). In addition, we found that retinol storage in the liver was significantly lower in MRL/lpr than MRL mice (S1A Fig.). Although the level of retinol in lupus-affected organs was not determined, we speculate that more retinol may have been depleted from the liver in order to facilitate immune response in other organs, thereby promoting lupus-like disease in MRL/lpr mice. Together, these results suggest that although tRA is able to improve glomerular pathology and reduce the size of lymph nodes in the mesenteric area, it has a generally deteriorating effect on peripheral inflammation when given after the initiation of disease in the MRL/lpr mouse model.

We observed a ∼50% reduction in the size of MLN in MRL/lpr mice (Fig. 4A), consistent with a previous report [27]. However, the proportion of B- and T-cell subsets did not change (Fig. 5B, S2B and S2C), suggesting normal differentiation and activation of lymphocytes with tRA treatment. Since tRA has been shown to induce apoptosis of cancer cells in the treatment of acute promyelocytic leukemia [61], the diminished lymphocyte numbers in MLN might be due to tRA-induced apoptosis. Another possibility is that tRA increased the trafficking of immune cells from MLN to peripheral tissues [62, 63]. The observation that tRA increased leukocyte infiltration in the brain, lung and kidney interstitium supported this hypothesis. Whether or not tRA regulated chemokine receptors CCR6, CCR4, and CX3CR1, which respectively regulate leukocyte migration to these tissues [64–66], required further investigation.

tRA is critical for the generation of Tregs under steady state [31]. Methods to enhance Tregs have been considered for several autoimmune diseases [67–70]. However, as shown in this study (S2D Fig.), tRA failed to induce peripheral Tregs under an immunogenic environment established early in lupus pathogenesis. In fact, when combined with proinflammatory cytokines such as IL-6, which was shown to be induced in tRA-treated MRL/lpr mice (S3A Fig.), tRA may facilitate the differentiation and activation of Th1 or Th17 cells instead of Tregs [2, 4]. The lack of Treg response upon tRA treatment may explain why tRA failed to induce systemic immunosuppression in MRL/lpr mice.

Two formulations were used in our study to administer vitamin A. One was a high dose of tRA alone, and the other was a low dose of tRA combined with retinyl palmitate (VARA). VARA was formulated with 10% of tRA, previously shown to enhance the esterification and storage of retinyl palmitate in the lung, and to a lesser extent, in the liver [49]. By comparing tRA to VARA treatment, we could potentially find out how different vitamin A metabolites affect autoimmunity. Because the concentration of tRA in the plasma and peripheral tissues is tightly regulated [71], even high doses of tRA can only sustain a high plasma level for a few hours, which is followed by dramatic decrease to the normal, physiological level [71–73]. However, tRA as a direct dose may be able to exert cellular functions faster than VARA, which requires time to generate tRA from retinyl palmitate. Indeed, our results showed that direct administration of tRA led to distinct changes in MRL/lpr mice that were not shared by VARA treatment (Table 1). In particular, while correlation analysis suggests that circulating autoantibodies detriment brain and lung pathology (Fig. 6), VARA increased blood levels of total and autoantibodies without exacerbating lupus-like disease in the brain and lung, indicating a possibly protective effect from this formulation. Together, these results suggest that different formulations of vitamin A may affect the immune system differently, and thus should be carefully considered if supplementation were to be used. It is worth mentioning that, for both tRA and VARA used in our study, the total amount of vitamin A was about 6 mg/kg body weight per day, which is 500× of Recommended Dietary Allowance for vitamin A and clearly a pharmacological dose, though liver toxicity was not observed (S1B Fig.).

It is worth mentioning that in human SLE, it has been well established that autoantibodies and the amount of IC deposits in the glomeruli are correlated with disease activity and in particular with lupus nephritis activity [74]. In our study of MRL/lpr mice, we observed increased anti-dsDNA IgG levels with tRA or VARA treatment, but that did not directly correlate with renal damages. This phenomenon might be due to the short lifespan of the mice and the fact that no single mouse model can mimic the complexity of human SLE [75]. Indeed, while another study in MRL/lpr mice observed a similar increase of autoantibodies with tRA treatment [27], in the NZBWF1 mouse model, tRA did not increase anti-dsDNA IgG titers [26, 28].

Together, the results of this study suggest that tRA may play paradoxical roles in SLE if it is given after an immunogenic environment has been established early in lupus pathogenesis. We showed that while treatment with tRA improved glomerular pathology, it also caused severe inflammation in other peripheral organs. The increased tissue inflammation may be associated with tRA-mediated augmentation of B-cell responses. Immunosuppressive Tregs, on the other hand, were not induced by tRA treatment in MRL/lpr mice. Paradoxically, tRA was able to shrink the lymph nodes in the mesenteric area by decreasing the number of lymphocytes, likely through promoting the trafficking of lymphocytes to the brain, lung, or kidney interstitium. In SLE patients, tRA combined with glucocorticoids has been shown to decrease the level of autoantibodies [24–27]. However, the effect of tRA alone on autoantibody production was not investigated. Nor was the effect of tRA on skin, lung, and brain of lupus patients. Our study in the MRL/lpr mice provides evidence that tRA may be harmful for lupus patients when administered alone. Although we analyzed younger mice without established clinical symptoms, we hypothesize that the effects of tRA on older mice with symptoms would be similar to those on younger ones, since the immunogenic microenvironment has already been established in younger mice and would be sustained when they get older. This hypothesis will be tested in the future. Nevertheless, our current observations suggest that further studies are necessary before a recommendation on the use of retinoid supplements can be made for SLE patients.

## Supporting Information

S1 Fig (A) Total retinol in the liver. (B) Liver function tests. Concentrations of alanine aminotransferase (ALT) and aspartate aminotransferase (AST) in the plasma of 14-week-old mice are shown. (C) Body weight. ns: not significant. Data are shown as mean ± SEM (n = 6 mice in each group). (D) The percentage of mice with (red) or without (blue) dermatitis on the back of the neck and/or face.
(TIF)

S2 Fig (A) Spleen weight, spleen/body weight ratio, and total number of cells in the spleen. ns: not significant, one-way ANOVA. (B) Percentages of CD4^+^CD8^-^, CD4^-^CD8^+^ and CD4^-^CD8^-^ in CD3^+^ T cells in the MLN as determined by flow cytometry. (C) Percentages of naïve T cells (CD62L^+^CD44^-^), central memory (CM) T cells (CD62L^+^CD44^+^) and effector memory (EM) T cells (CD62L^-^CD44^+^) in CD3^+^CD4^+^Foxp3^-^CD25^-^ (non-Treg CD4^+^) T cells in the MLN. (D) Percentages and absolute numbers of Tregs (CD3^+^CD4^+^Foxp3^+^CD25^+^) in the spleen and MLN. (E) Percentages of pDC (CD11b^-^CD11c^+^Siglec-H^+^B220^+^), CD11b^+^ cDC (B220^-^CD11c^+^CD11b^+^MHC-II^+^), and CD11b^-^ cDC (CD11b^-^Siglec-H^-^B220^-^CD11c^+^MHC-II^+^) in the MLN. ns: not significant, * P<0.05, ** P<0.01, *** P<0.001, one-way ANOVA; +: P<0.05, student’s *t*-test. Data are shown as mean + SEM or mean ± SEM (n = 6 mice in each group).
(TIF)

S3 Fig (A) mRNA levels of IL-6, IL-21 and IFN in the MLN at 14 weeks old as determined by RT-qPCR. Relative quantities (RQ, log scale) of cytokine mRNA were normalized to that of L32. The average log RQ value of MRL vehicle group was defined as 1. (B) Correlation analysis between blood autoantibody levels and pathological scores with the outlier included (shown in green).
(TIF)

S4 Fig (A) The absolute cell numbers of plasma cells (CD19^-^CD27^-^CD138^+^CD44^+^) and plasmablasts (CD19^+/low^CD27^+/low^CD138^+^CD44^+^) in the spleen and MLN at 14 weeks old as measured by flow cytometry. (B) Percentages of naïve T cells (CD62L^+^CD44^-^), central memory (CM) T cells (CD62L^+^CD44^+^) and effector memory (EM) T cells (CD62L^-^CD44^+^) in CD3^+^CD4^+^Foxp3^-^CD25^-^ (non-Treg CD4^+^) T cells, and percentages of Tregs (CD3^+^CD4^+^Foxp3^+^CD25^+^) in CD3^+^ T cells in the spleen and MLN at 14 weeks old as measured by flow cytometry. ns: not significant, * P<0.05, ** P<0.01, student’s *t*-test. Data are shown as mean ± SEM (n = 3 mice in each group).
(TIF)

S1 Primer Sequences (PDF)
