# Supplementary figures and images for: Paradoxical Effects of All-Trans-Retinoic Acid on Lupus-Like Disease in the MRL/lpr Mouse Model
Source: PLoS One. 2015 Mar 16;10(3):e0118176. doi: 10.1371/journal.pone.0118176 (PMC4361690; doi:10.1371/journal.pone.0118176)

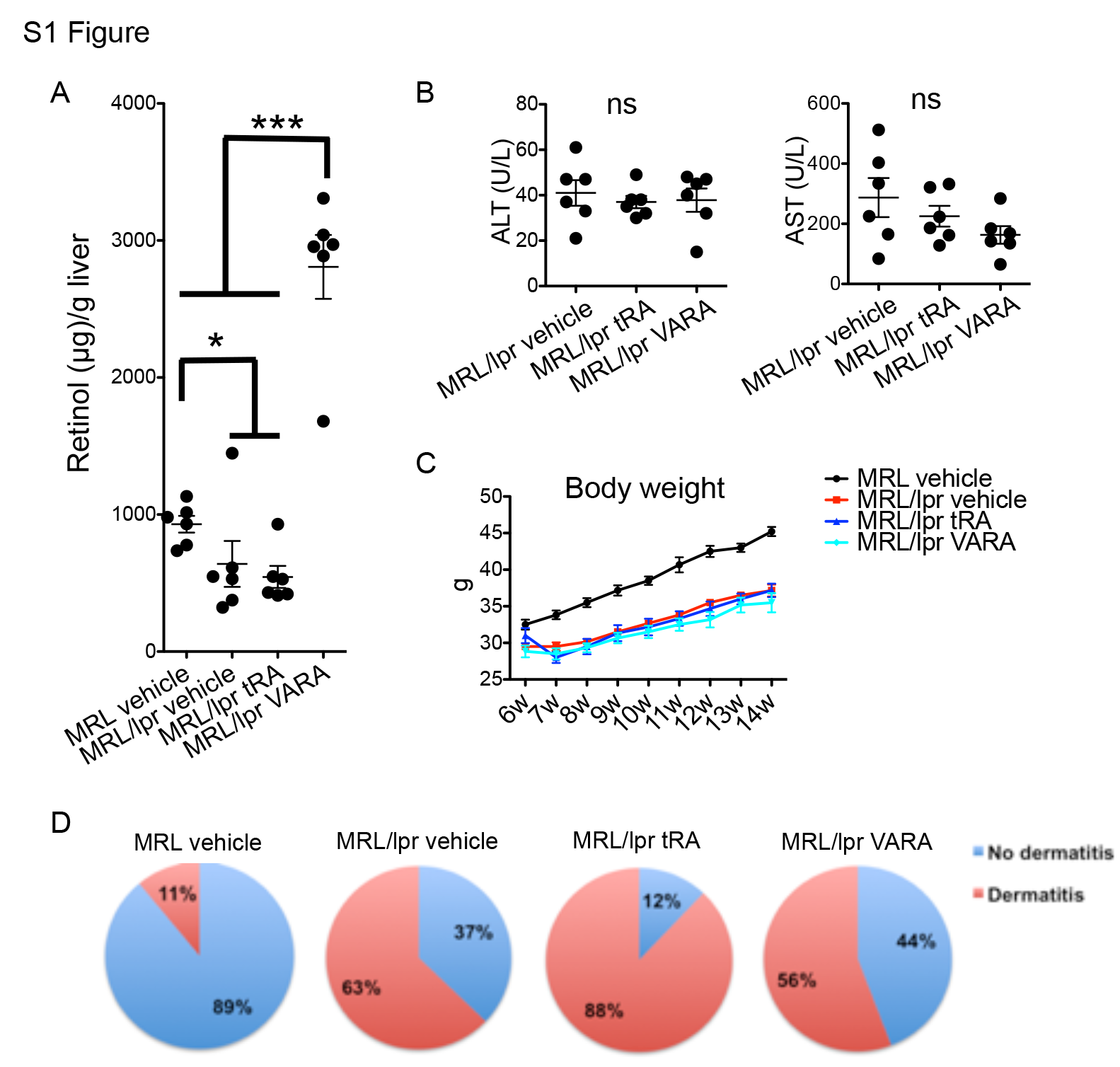

Supplement: S1 Fig — (B) Liver function tests. Concentrations of alanine aminotransferase (ALT) and aspartate aminotransferase (AST) in the plasma of 14-week-old mice are shown. (C) Body weight. ns: not significant. Data are shown as mean ± SEM (n = 6 mice in each group). (D) The percentage of mice with (red) or without (blue) dermatitis on the back of the neck and/or face. (TIF) [file pone.0118176.s001.tif]

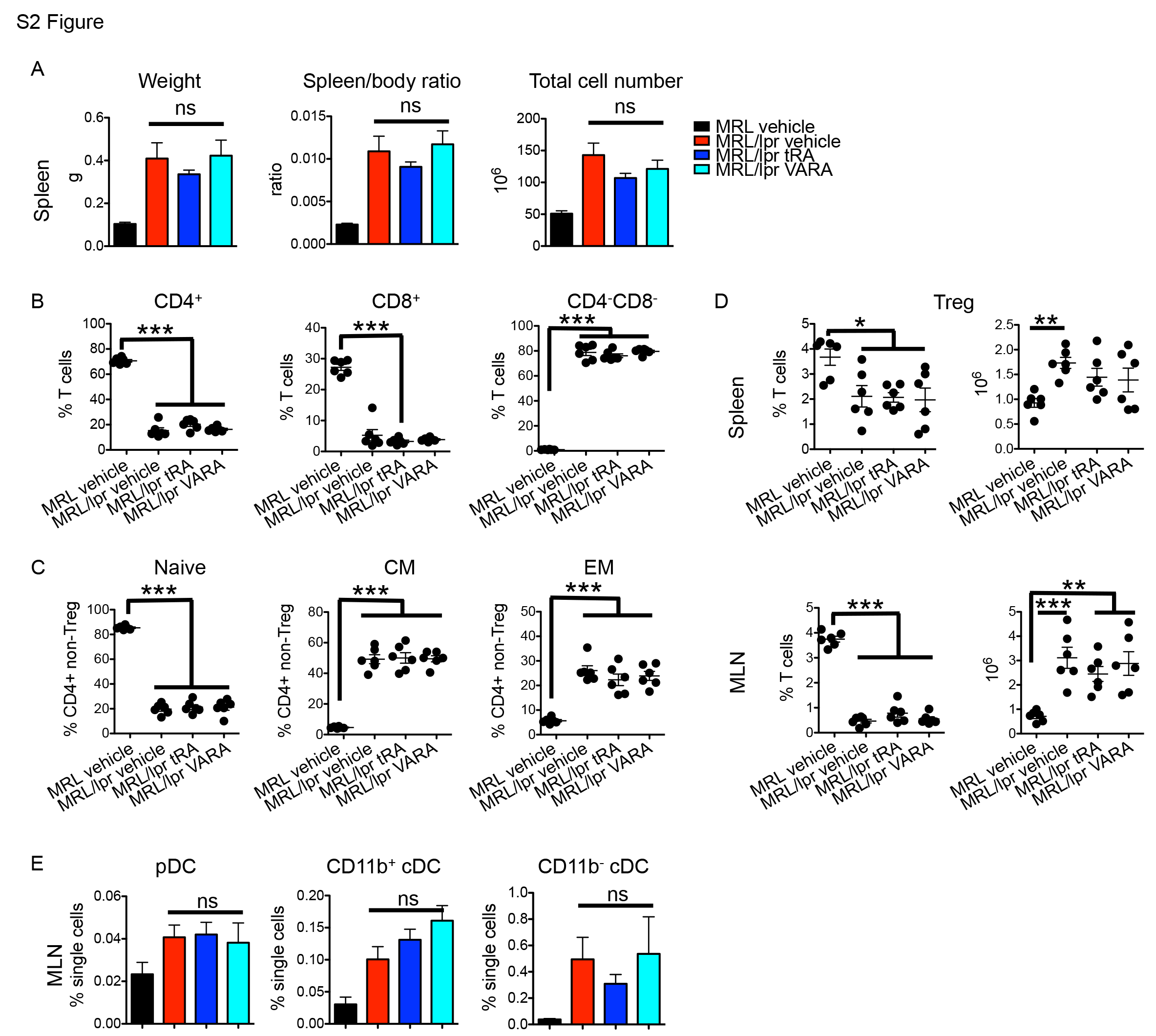

Supplement: S2 Fig — ns: not significant, one-way ANOVA. (B) Percentages of CD4+CD8-, CD4-CD8+ and CD4-CD8- in CD3+ T cells in the MLN as determined by flow cytometry. (C) Percentages of naïve T cells (CD62L+CD44-), central memory (CM) T cells (CD62L+CD44+) and effector memory (EM) T cells (CD62L-CD44+) in CD3+CD4+Foxp3-CD25- (non-Treg CD4+) T cells in the MLN. (D) Percentages and absolute numbers of Tregs (CD3+CD4+Foxp3+CD25+) in the spleen and MLN. (E) Percentages of pDC (CD11b-CD11c+Siglec-H+B220+), CD11b+ cDC (B220-CD11c+CD11b+MHC-II+), and CD11b- cDC (CD11b-Siglec-H-B220-CD11c+MHC-II+) in the MLN. ns: not significant, * P<0.05, ** P<0.01, *** P<0.001, one-way ANOVA; +: P<0.05, student’s t-test. Data are shown as mean + SEM or mean ± SEM (n = 6 mice in each group). (TIF) [file pone.0118176.s002.tif]

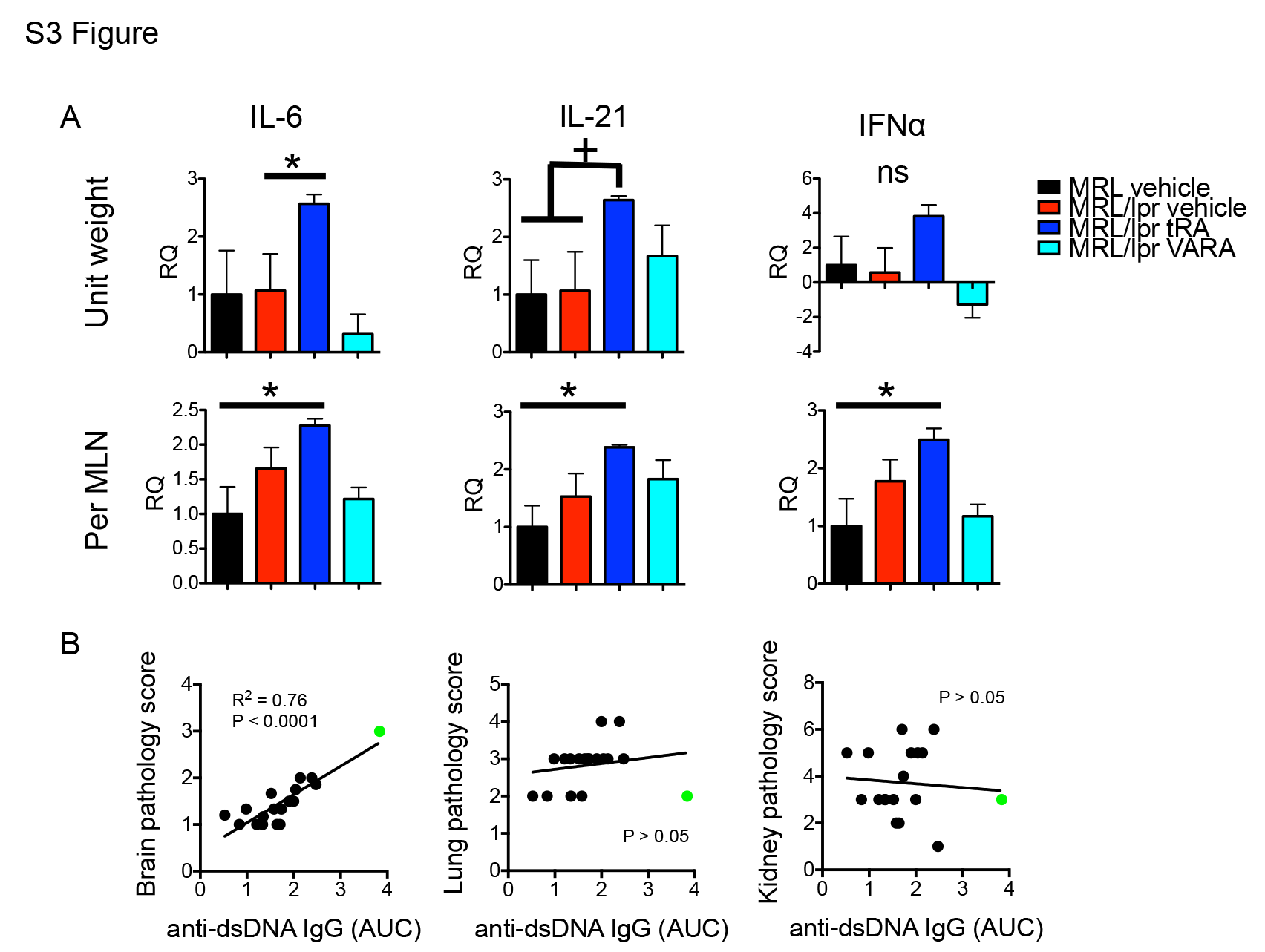

Supplement: S3 Fig — Relative quantities (RQ, log scale) of cytokine mRNA were normalized to that of L32. The average log RQ value of MRL vehicle group was defined as 1. (B) Correlation analysis between blood autoantibody levels and pathological scores with the outlier included (shown in green). (TIF) [file pone.0118176.s003.tif]

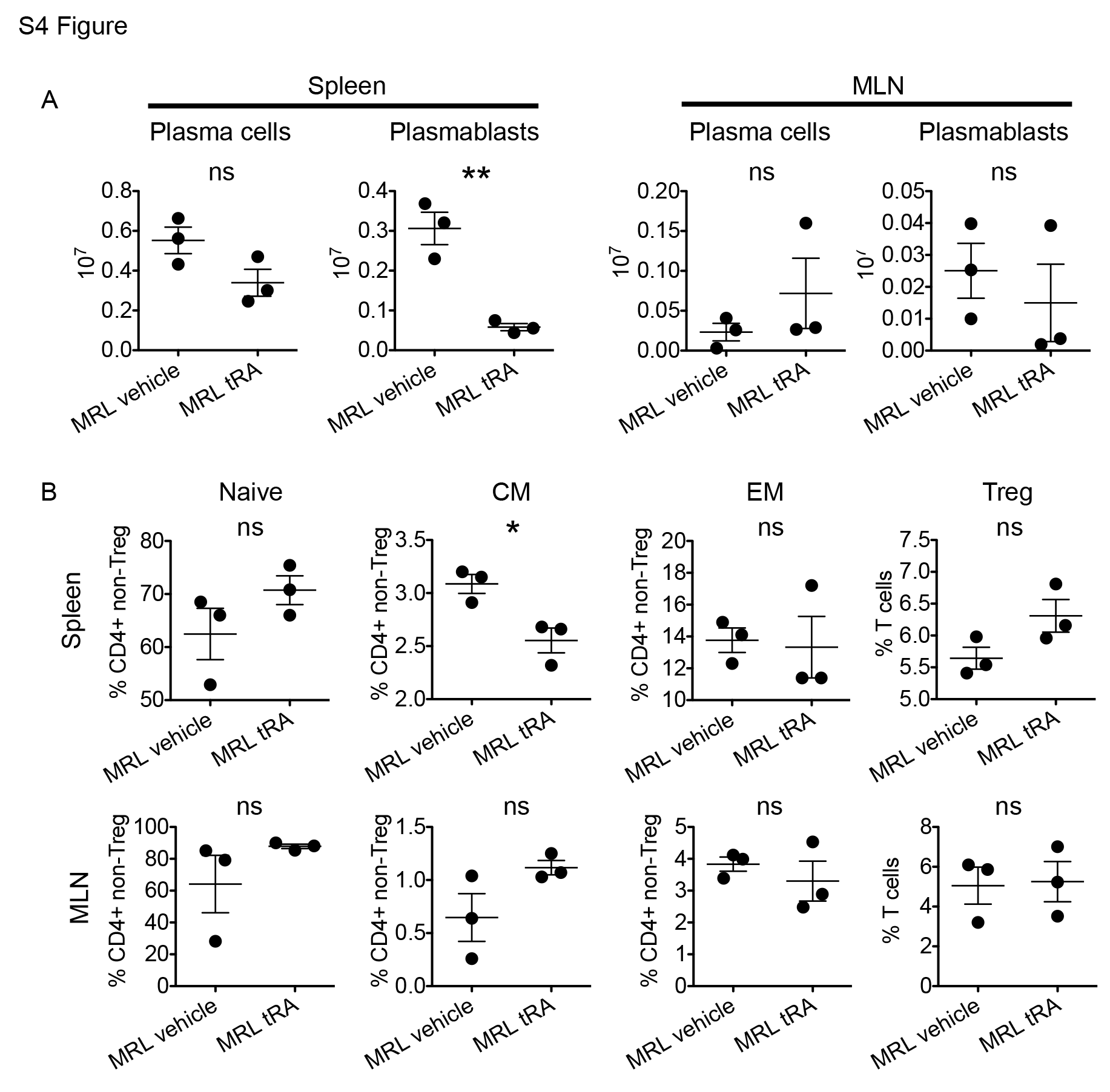

Supplement: S4 Fig — (B) Percentages of naïve T cells (CD62L+CD44-), central memory (CM) T cells (CD62L+CD44+) and effector memory (EM) T cells (CD62L-CD44+) in CD3+CD4+Foxp3-CD25- (non-Treg CD4+) T cells, and percentages of Tregs (CD3+CD4+Foxp3+CD25+) in CD3+ T cells in the spleen and MLN at 14 weeks old as measured by flow cytometry. ns: not significant, * P<0.05, ** P<0.01, student’s t-test. Data are shown as mean ± SEM (n = 3 mice in each group). (TIF) [file pone.0118176.s004.tif]
