# Supplementary material for: Paradoxical Effects of All-Trans-Retinoic Acid on Lupus-Like Disease in the MRL/lpr Mouse Model
Source: PLoS One. 2015 Mar 16;10(3):e0118176. doi: 10.1371/journal.pone.0118176 (PMC4361690; doi:10.1371/journal.pone.0118176)
Supplement: S1 Primer Sequences — (PDF) [file pone.0118176.s005.pdf]

S1 Primer Sequences

IL-6 forward:	5'-TAG TCC TTC CTA CCC CAA TTT CC-3'
IL-6 backward:	5'- TTG GTC CTT AGC CAC TCC TTC-3'
IL-21 forward:	5'-GGA CCC TTG TCT GTC TGG TAG-3'
IL-21 backward:	5'-TGT GGA GCT GAT AGA AGT TCA GG-3'
IFNα forward:	5'-TGA TGA GCT ACT ACT GGT CAG C-3'
IFNα backward:	5'-GAT CTC TTA GCA CAA GGA TGG C-3'
L32 forward:	5'-AAG CGA AAC TGG CGG AAA C-3'
L32 backward:	5'-TAA CCG ATG TTG GGC ATC AG-3'
